# Prevalence and determinants of symptomatic COVID-19 infection among children and adolescents in Qatar: a cross-sectional analysis of 11 445 individuals

**DOI:** 10.1017/S0950268821001515

**Published:** 2021-07-02

**Authors:** Omran A. H. Musa, Tawanda Chivese, Devendra Bansal, Jazeel Abdulmajeed, Osman Ameen, Nazmul Islam, Chang Xu, Mohamed A. Sallam, Soha S. Albayat, Hayat S. Khogali, Shazia N. N. Ahmed, Sayed M. Himatt, Mohamed Nour, Aiman A. Elberdiny, Abdallah Musa, Luis Furuya-Kanamori, Hamad E. Al-Romaihi, Suhail A. R. Doi, Mohammed H. J. Al-Thani, Elmoubashar Abu Baker Abd Farag

**Affiliations:** 1 Department of Population Medicine, College of Medicine, Qatar University, Doha, Qatar; 2 Department of Public Health, Ministry of Public Health, Doha, Qatar; 3 Department of Strategy Planning & HI – Business & Health Intelligence, Primary Health Care Corporation, Doha, Qatar; 4 Department of Clinical Science, College of Medicine, Qatar University, Doha, Qatar; 5 Department of Public Health, College of Health Sciences, Qatar University, Doha, Qatar; 6 Department of Basic Medical Science, College of Medicine, Qatar University, Doha, Qatar; 7 Faculty of Medicine, QU Centre for Clinical Research, The University of Queensland, Herston, Australia

**Keywords:** Children, Qatar, symptomatic COVID-19

## Abstract

There is a paucity of evidence about the prevalence and risk factors for symptomatic infection among children. This study aimed to describe the prevalence of symptomatic coronavirus disease 2019 (COVID-19) and its risk factors in children and adolescents aged 0–18 years in Qatar. We conducted a cross-sectional study of all children aged 0–18 years diagnosed with COVID-19 using polymerase chain reaction in Qatar during the period 1st March to 31st July 2020. A generalised linear model with a binomial family and identity link was used to assess the association between selected factors and the prevalence of symptomatic infection. A total of 11 445 children with a median age of 8 years (interquartile range (IQR) 3–13 years) were included in this study. The prevalence of symptomatic COVID-19 was 36.6% (95% confidence interval (CI) 35.7–37.5), and it was similar between children aged <5 years (37.8%), 5–9 years (34.3%) and 10 + years (37.3%). The most frequently reported symptoms among the symptomatic group were fever (73.5%), cough (34.8%), headache (23.2%) and sore throat (23.2%). Fever (82.8%) was more common in symptomatic children aged <5 years, while cough (38.7%) was more prevalent in those aged 10 years or older, compared to other age groups. Variables associated with an increased risk of symptomatic infection were; contact with confirmed cases (RD 0.21; 95% CI 0.20–0.23; *P* = 0.001), having visited a health care facility (RD 0.54; 95% CI 0.45–0.62; *P* = 0.001), and children aged under 5 years (RD 0.05; 95% CI 0.02–0.07; *P* = 0.001) or aged 10 years or older (RD 0.04; 95% CI 0.02–0.06; *P* = 0.001). A third of the children with COVID-19 were symptomatic with a higher proportion of fever in very young children and a higher proportion of cough in those between 10 and 18 years of age.

## Introduction

Since its discovery in December 2019, the coronavirus disease 2019 (COVID-19) remains one of the biggest global health challenges. To date, more than 38 million people have been infected by the severe acute respiratory syndrome coronavirus 2 (SARS-CoV-2) which causes COVID-19 and more than 1 million people have died as a result of infection [1]. Children comprise roughly 10% of all the individuals infected with COVID-19 [2]. In Qatar, since the beginning of the pandemic in February 2020 up to April 2021, over 180 000 people have been confirmed to be infected with COVID-19, of whom a minority were children (aged 0–18 years old) [3]. As in other countries, there was a need in Qatar to define symptomatology in children to develop and implement school health policies and determine what constitutes a clinical case definition [4].

Although research suggests that children have a lower susceptibility to COVID-19 infection [5–7] compared to adults, children can still get infected by the virus, and are capable of transmitting the virus to others [8]. Research also suggests that children are less likely to develop symptomatic infection and less likely to have severe disease, compared to adults [6, 9]. Children have been indirectly affected by COVID-19 through many other ways such as through school closures in many countries [10–12]. The controversy about return-to-school policies remains unresolved though there is increasing evidence that transmission dynamics in children differed from that in adults [13, 14].

A first step towards understanding COVID-19 dynamics in children is to understand the nature of symptomatic infection in children, the predominant symptoms and how symptomatic infection differs by age [15]. Available evidence to date has mainly been from China, where the pandemic is largely controlled [16]. The prevalence of symptomatic infection in children was estimated to range between 9.7 and 27.3% [17]. Research suggests that infants less than 1 year of age have an increased risk of symptomatic infection, compared to other age groups, although this observation is not based on large representative studies [18, 19]. Male children have also been found to be more likely to have asymptomatic infection, although these results are based on a systematic review of 158 cases only [15]. Overall, the determinants of symptomatic infection are poorly understood. Therefore, in this study, we sought to estimate the prevalence of symptomatic infection and to describe the most common symptoms of COVID-19 in children with symptomatic infection and to investigate associated risk factors for symptomatic infection in children and adolescents aged 0–18 years.

## Methods

### Design and setting

A cross-sectional study of all children diagnosed with COVID-19 in Qatar during the period between 1st March and 31st July 2020 was carried out. Data on demographic information and clinical characteristics were obtained from the Ministry of Public Health (MoPH), Qatar database. The national surveillance system ‘Surveillance and Vaccination Electronic System’ (SAVES) at the MoPH receives notifications from all healthcare facilities across the country, mainly from Hamad Medical Corporation (HMC) health facilities and Primary Health Care Corporation (PHCC). HMC and PHCC are the main non-profit public health care providers that manage 10 highly specialised hospitals, 27 PHCC health centres, as well as other governmental and semi-governmental health institutions. Children included in this study were consecutive cases tested either because they were symptomatic, contacts of other cases or if they were returned citizens or residents from travel. Trained health professional volunteers collected the data through a structured COVID-19 case investigation form developed by the MoPH based on best evidence that is currently available (WHO protocol) [20].

### Study population

Children subjects diagnosed with COVID-19 aged 0–18 years old with laboratory confirmed SARS-COV-2 infection (i.e. PCR positive on nasopharyngeal swab) status between 1st March and 31st July 2020 were eligible for inclusion in the study.

### Assessment of COVID-19 symptoms

Once the confirmed COVID-19 case was entered into the MOPH notification system, active case investigation was conducted by the MoPH surveillance team within 24–48 h using the case investigation form. Children were assessed for fever, cough, shortness of breath or difficulty breathing during the case investigations (children or parents were interviewed). In addition, for each case the team also collected data on age, nationality (Qatari/non-Qatari), ethnicity and clinical history. In addition, symptomatic infection was defined as the presence of any of a pre-defined list of possible symptoms during infection.

### Ethics approval

Ethics approval (ERC-826-3-2020) and waiver of informed consent were given by the Health Research Governance Department at the MoPH. All data were deidentified before analysis.

### Statistical analysis

Descriptive analyses were done for the presentation of baseline characteristics of the participants. Variables were summarised by the mean and standard deviation or median and interquartile range (IQR) based on data characteristics. The *t*-test or the Wilcoxon rank-sum test were applied to continuous variables while chi-square test was applied to categorical variables for differences between groups.

Age was categorised into three categories (<5, 5–9 and 10+ years old). Nationality was first grouped into Qatari *vs.* non-Qatari, and then it was further divided into seven ethnic categories based on geographical location: Middle East and North Africa (MENA), Asia, Africa, North America, Europe, South America and Oceania (Supplementary material).

A generalised linear model with a binomial family and identity link was used to assess the association between symptomatic infection and selected factors, including age, gender, nationality and mode of transmission (contact with a confirmed case in the last 14 days or visited a health facility within the last 14 days). The linktest was used to test model specification. The exact *P*-values were reported, and Stata MP version 15 (StataCrop, College Station, TX, USA) was utilised for all the analyses.

## Results

A total of 11 445 children with COVID-19 were included in the analysis, summarised in Table 1. The sample had a median age of 8 years (IQR 3–13) and the distribution of children across age groups was almost one third in children aged < 5 years (30.9%) and 5–9 years (29.0%), and it was (40.1%) in children 10 years or older of the total. Amongst the total cases, approximately two thirds (66.7%) of the children infected with COVID-19 were non-Qatari and half (50.6%) of the children were males. Two thirds (66.0%) of the infected children had confirmed contact with a case diagnosed with COVID-19 within the last 14 days. In addition, there were no deaths reported among children in the sample.

**Table 1.** Demographic characteristics of children with COVID-19 by symptomatic status^a^

	Total *N* = 11 445	Symptomatic *N* = 4187	Asymptomatic *N* = 7258	*P*-value
Age (years)	8 (3, 13)	8 (3, 13)	8 (4, 12)	0.357
Age categories				
<5	3531 (30.9)	1334 (31.9)	2197 (30.3)	0.006
5–9	3318 (29.0)	1138 (27.2)	2180 (30.0)	
10+	4596 (40.1)	1715 (41.0)	2881 (39.7)	
Nationality^b^				
Non-Qatari	7633 (66.7)	2935 (70.1)	4398 (64.7)	<0.001
Qatari	3808 (33.3)	1251 (29.9)	2557 (35.2)	
Ethnic group^b^				
MENA	7539 (65.9)	2623 (62.7)	4916 (67.7)	<0.001
Asian	3681 (32.2)	1484 (35.4)	2197 (30.3)	
North American	99 (0.9)	44 (1.1)	55 (0.8)	
African	60 (0.5)	17 (0.4)	43 (0.6)	
European	50 (0.4)	16 (0.4)	34 (0.5)	
South American	8 (0.1)	1 (0.02)	7 (0.1)	
Oceanian	4 (0.03)	1 (0.02)	3 (0.04)	
Gender				
Male	5795 (50.6)	2076 (49.6)	3719 (51.2)	0.087
Female	5650 (49.4)	2111 (50.4)	3539 (48.8)	
Mode of transmission				
Contact with confirmed case in the last 14 days	7556 (66.0)	3295 (78.7)	4261 (58.7)	<0.001
Visited a healthcare facility in the last 14 days	580 (5.1)	342 (8.2)	238 (3.3)	<0.001
Travelled in the last 14 days	62 (0.5)	22 (0.5)	40 (0.6)	0.857

a Due to rounding percentages might not add up to 100%. Median (IQR) is reported for continuous data and *n* (%) for categorical data.

b Four nationalities were unknown.

**Fig. 1.**
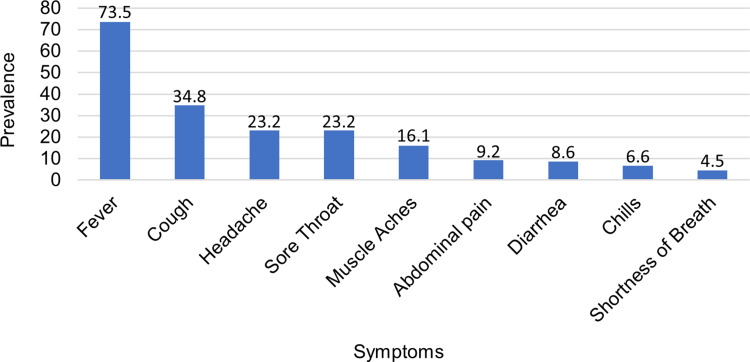
Prevalence of symptoms in symptomatic children with COVID-19.

**Fig. 2.**
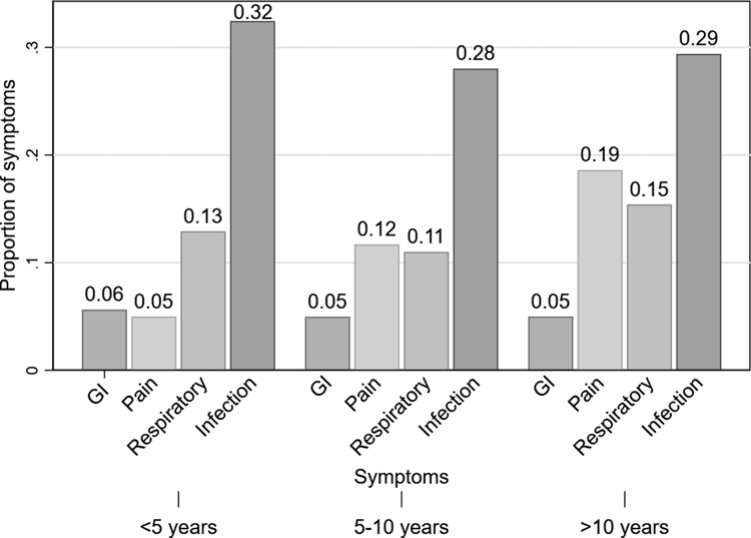
Proportion of symptoms by age groups.

The prevalence of symptomatic COVID-19 was 36.6% (95% CI 35.7–37.5), and it was similar in children aged <5 years (37.8%), 5–9 years (34.3%) and 10 years or older (37.3%). Moreover, regarding the prevalence of clinical manifestations in children with symptomatic infection, fever (73.5%) and cough (34.8%) were the most prevalent symptoms, whereas chills (6.6%) and shortness of breath (4.5%) were less common (Fig. 1). Similarly, across the entire age groups, fever and cough were the most common symptoms, while shortness of breath was the least prevalent. In particular, fever (82.8%) was more common in symptomatic children aged <5 years, while cough (38.7%) was more prevalent in those aged 10 years or older, compared to other age groups (Table 2). When clustered by type of symptom, only pain (headache, musculoskeletal pain and abdominal pain) increased with age, whereas gastrointestinal (GI), respiratory and other infection-related symptoms were similar across all age groups (Fig. 2).

**Table 2.** Comparison of symptoms in 4187 children with symptomatic COVID-19 by age group

	Total *N* = 4187	<5 years *N* = 1334	5–9 years *N* = 1138	10 + years *N* = 1715	*P*-value
Abdominal pain	386 (9.2)	79 (5.9)	129 (11.3)	178 (10.4)	<0.001
Chills	278 (6.6)	62 (4.6)	73 (6.4)	143 (8.3)	0.001
Cough	1455 (34.8)	442 (33.1)	349 (30.7)	664 (38.7)	<0.001
Diarrhoea	360 (8.6)	165 (12.4)	77 (6.8)	118 (6.9)	<0.001
Fever	3078 (73.5)	1104 (82.8)	842 (74.0)	1132 (66.0)	<0.001
Headache	973 (23.2)	63 (4.7)	259 (22.8)	651 (38.0)	<0.001
Muscle aches	673 (16.1)	82 (6.1)	163 (14.3)	428 (25.0)	<0.001
Shortness of breath	187 (4.5)	44 (3.3)	34 (3.0)	109 (6.4)	<0.001
Sore throat	970 (23.2)	182 (13.6)	256 (22.5)	532 (31.0)	<0.001

Multivariable analysis (Table 3) revealed that the baseline symptomatic proportion in children with no contact history, no visit to the health care facility, of Qatari status and of age 5–9 years was 15.0%. Children aged below 5 and 10–18 years had an increased symptomatic proportion of 5.0% [risk difference (RD) 0.05; 95% CI 0.02–0.07; *P* < 0.001] and 4.0% (RD 0.04; 95% CI 0.02–0.06; *P* < 0.001) respectively compared to age 5–9 years. Non-Qatari children also had an increased symptomatic proportion of 5.0% (RD 0.05; 95% CI 0.04–0.07; *P* < 0.001) compared to Qatari children. Both contact with a confirmed case (RD 0.21; 95% CI 0.20–0.23; *P* < 0.001) and visit to a health care facility (RD 0.54; 95% CI 0.45–0.62; *P* < 0.001) were associated with a markedly increased symptomatic proportion compared to those without a contact history. However, there was an antagonistic interaction between the latter such that both contact with a confirmed case and visit to a health facility together decreased symptomatic proportions by 41.0% over the expected additive increases associated with both exposures.

**Table 3.** Factors associated with symptoms in children with COVID-19^a^

	RD	95% CI	*P*-value
Age categories			
0 to <5 years	0.05	0.02–0.07	<0.001
10+ years	0.04	0.02–0.06	<0.001
Nationality			
Non-Qatari	0.05	0.04–0.07	<0.001
Gender			
Female	0.00	−0.02 to 0.02	0.867
Mode of transmission			
Contact with a confirmed case in the last 14 days	0.21	0.20–0.23	<0.001
Visit health facility within the last 14 days	0.54	0.45–0.62	<0.001
Interaction (contact -Yes and visit-Yes)	−0.41	−0.51 to −0.31	<0.001

a Generalised linear model with binomial family, identity link and RD as the effect measure. Children aged 5–9 years, Qatari, males, no-contact and no-visit were the reference groups with baseline risk of being symptomatic being 0.15 (constant in the regression model). The RDs in this analysis are not transferable beyond the data presented here, however, the trend can be generalised to other paediatric populations.

## Discussion

We found that almost one-third of children with COVID-19 infection were symptomatic, in a large cohort of children aged from the first year of life through to the age of 18 years. Almost three-quarters of the children with symptomatic COVID-19 infection had fever, and perhaps this could be due to a higher viral load while cough was the second most commonly reported symptom, but with just over one-third of children reporting this. In addition, pain was mainly limited to older children, perhaps children in this age group are better to communicate this symptom. Children of ages <5 (31.9%) and 10+ years (41.0%) had a higher risk of being symptomatic (these are higher percentages than reported in Table 3 because they include children with contact history). Indeed, contact with a confirmed COVID-19 case, and visiting a health facility in the 2 weeks prior to the diagnosis of COVID-19 significantly increased the risk of having symptomatic COVID-19 infection, which could be explained as an effect−cause situation.

In the current study, the prevalence of symptoms in low-risk children (no contact history, no visit to a health care facility, Qatari status and age 5–9 years) was 15.0%. The prevalence of COVID-19 symptoms was found to be about double of this baseline in the whole population of children (36.6%). However, several studies contradict this finding [19, 21–23]. For example, Hoang *et al*. reported around 80.0% prevalence of symptomatic infection across 131 studies [23]. Similarly, another meta-analysis study showed 84.0% of symptomatic infection among infected children [24]. This disparity could be a consequence of very small sample sizes and selection of sick children into previous studies.

Prevailing symptoms in children with symptomatic COVID-19 was topped by fever (73.5%) and was followed by cough (34.8%). This was also consistent with several reports [22–32]. For instance, in a study of 7780 children with COVID-19, fever (59.1%) and cough (55.9%) were the symptoms most frequent reported [23]. Moreover, Giacomet *et al*. also found fever (79.4%) and cough (48.6%) more common [30]. Shortness of breath was the least prevalent symptom (4.5%) reported in this study, and the finding was contrary to some previous reports [19, 21, 22, 24, 26, 33]. For example, abdominal pain (8%) was the least frequent in a meta-analysis of 1614 infected children [24]. A retrospective study conducted by Lu *et al*. also reported musculoskeletal symptoms (2.7%) were least common in 110 children with COVID-19 [19]. Another review study also found chest pain (0.4%) to be least prevalent [33]. This discrepancy could have several reasons. Firstly, two of the studies reporting symptoms were meta-analyses [22, 24] and one was a review [33] and thus heterogeneity of results is expected. Secondly, two studies included subjects with comorbidities [21, 26], which might be a source of variation among symptoms reported as children with chronic conditions are more likely to get severely ill. In addition, rather than depend solely on the database, in this study the symptomatic status was ascertained through phone calls with the parents.

This study found that children of less than 5 years and 10 or more years of age were more susceptible to symptomatic COVID-19 infection, compared to children aged 5–9 years. Similar findings were also reported in China [19, 34] but so far the exact reasons behind this phenomenon remain unknown.

The current study indicated approximately two thirds of the children infected had a contact with a COVID-19 confirmed case (66.0%) but very few had visited a health care facility (about 5.0%), which is consistent with several previous studies [27, 29, 35]. For instance, Qiu *et al*. reported that the majority of the children infected had a history of exposure to a family member with confirmed status of COVID-19 [27]. In addition, a meta-analysis of nine case series indicated that 75.0% of the infected children had a direct contact with a COVID-19 case [22]. Another study reported that 63.0% out of 68 infected children had contact with a confirmed case [21]. In this study, symptomatic status was 21.0% more in those with contact history and 54.0% more in those with health system visits. However, when both occurred together, the expected increase should have been 75.0% (54 + 21%) but due to the antagonistic interaction this was only 34.0% (75–41%; see Table 3) suggesting that many patients with both contact and a health system visit were asymptomatic.

The main strength of this study, is that it is the largest population-based study of COVID-19 infection among children to date. This study also has some limitations. Firstly, this study was a cross-sectional study, which does not assess causal relationship, but rather generates hypotheses. Secondly, our study was limited to available MoPH data. Data on comorbidities and hospitalisation need further study to gain further insights on the evolution of symptoms and clinical course of COVID-19 infection in children.

In conclusion, a third of the children with COVID-19 are symptomatic with a higher proportion of symptomatic infection in children at the two extremes of age. Fever is the predominant symptom in symptomatic children and a clear contact in the community or a visit to hospital markedly increases the possibility of a symptomatic infection.

## Data Availability

The data that support the findings will be provided upon request.

## Supplementary material

For supplementary material accompanying this paper visit http://dx.doi.org/10.1017/S0950268821001515. click here to view supplementary material
