# Prevalence and determinants of symptomatic COVID-19 infection among children and adolescents in Qatar: a cross-sectional analysis of 11 445 individuals

**DOI:** 10.1017/S095026882100203X

**Published:** 2021-09-14

**Authors:** Omran A. H. Musa, Tawanda Chivese, Devendra Bansal, Jazeel Abdulmajeed, Osman Ameen, Nazmul Islam, Chang Xu, Mohamed A. Sallam, Soha S. Albayat, Hayat S. Khogali, Shazia N. N. Ahmed, Sayed M. Himatt, Mohamed Nour, Aiman A. Elberdiny, Abdallah Musa Abdallah, Luis Furuya-Kanamori, Hamad E. Al-Romaihi, Suhail A. R. Doi, Mohammed H. J. Al-Thani, Elmoubashar Abu Baker Abd Farag

**Affiliations:** 1 Department of Population Medicine, College of Medicine, Qatar University, Doha, Qatar; 2 Department of Public Health, Ministry of Public Health, Doha, Qatar; 3 Department of Strategy Planning & HI–Business & Health Intelligence, Primary Health Care Corporation, Doha, Qatar; 4 Department of Clinical Science, College of Medicine, Qatar University, Doha, Qatar; 5 Department of Public Health, College of Health Sciences, Qatar University, Doha,Qatar; 6 Department of Basic Medical Science, College of Medicine, Qatar University, Doha, Qatar; 7 Faculty of Medicine, QU Centre for Clinical Research, The University of Queensland, Herston, Australia

The original version of this article was published with a mistake in one of the authors’ names. “Abdallah Musa Abdallah” is the correct full name but this was initially published as just “Abdallah Musa”. This has now been corrected.
