# Supplementary material for: Prevalence and determinants of symptomatic COVID-19 infection among children and adolescents in Qatar: a cross-sectional analysis of 11 445 individuals
Source: Epidemiol Infect. 2021 Jul 2;149:e193. doi: 10.1017/S0950268821001515 (PMC8387684; doi:10.1017/S0950268821001515)
Supplement: Supplementary file 1 [file S0950268821001515sup001.docx]

**Supplementary materials**

Middle East and North Africa (MENA) (Algeria, Bahraini, Cypriot/turkey, Egyptian, Emirati, Iranian, Iraq, Jordanian, Kuwaiti, Lebanese, Libyan, Mauritanian, Moroccan, Omani, Palestinian, Qatari, Saudi, Somali, Sudanese, Syrian, Tunisian, Turkish, and Yemeni), Asia (Afghanistan, Armenia, Bangladeshi, Chinese, Pilipino, Indian, Indonesian, Malaysian, Nepalese, Pakistani, South Korean, Srilankan, Uzbekistani, and Vietnamese), Africa (Beninese, Eritrean, Ethiopian, Ghanaian, Kenyan, Nigerian, South African, and Zimbabwean), North America (American, Belizean, and Canadian), Europe (Belorussia, Belgium, British, Danish, Dominican, Finnish, French, German, Greek, Hungarian, Irish, Italian, Moldovan, Romanian, Russia, Serbia, Spanish, and Swedish), South America (Brazilian and Ecuadorian) and Oceania (Australian and New Zealander)
